# An Antigenic Space Framework for Understanding Antibody Escape of SARS-CoV-2 Variants

**DOI:** 10.3390/v13102009

**Published:** 2021-10-06

**Authors:** Nathaniel L. Miller, Thomas Clark, Rahul Raman, Ram Sasisekharan

**Affiliations:** 1 Harvard-MIT Division of Health Sciences and Technology, Massachusetts Institute of Technology, Cambridge, MA 02139, USA; nkmiller@mit.edu; 2 Department of Biological Engineering, Massachusetts Institute of Technology, Cambridge, MA 02139, USA; 3 Koch Institute for Integrative Cancer Research, Massachusetts Institute of Technology, Cambridge, MA 02139, USA; clarkt@mit.edu (T.C.); rraman@mit.edu (R.R.)

**Keywords:** SARS-CoV-2, epitope, paratope, antibody, immune escape, variant, VOC, antigenic drift

## Abstract

The evolution of mutations in SARS-CoV-2 at antigenic sites that impact neutralizing antibody responses in humans poses a risk to immunity developed through vaccination and natural infection. The highly successful RNA-based vaccines have enabled rapid vaccine updates that incorporate mutations from current variants of concern (VOCs). It is therefore important to anticipate future antigenic mutations as the virus navigates the heterogeneous global landscape of host immunity. Toward this goal, we survey epitope-paratope interfaces of anti-SARS-CoV-2 antibodies to map an antigenic space that captures the role of each spike protein residue within the polyclonal antibody response directed against the ACE2-receptor binding domain (RBD) or the N-terminal domain (NTD). In particular, the antigenic space map builds on recently published epitope definitions by annotating epitope overlap and orthogonality at the residue level. We employ the antigenic space map as a framework to understand how mutations on nine major variants contribute to each variant’s evasion of neutralizing antibodies. Further, we identify constellations of mutations that span the orthogonal epitope regions of the RBD and NTD on the variants with the greatest antibody escape. Finally, we apply the antigenic space map to predict which regions of antigenic space—should they mutate—may be most likely to complementarily augment antibody evasion for the most evasive and transmissible VOCs.

## 1. Introduction

Since the emergence of the coronavirus SARS-CoV-2 in 2019, SARS-CoV-2 has evolved within the context of a complex global landscape of diverse human host immunity arising from varied infections and vaccinations. The continuing adaptation of the virus has resulted in several variants of concern (VOC) that are more transmissible [1], virulent [2], and partially resistant to antibodies [3] and immunity acquired through convalescence [4] and vaccination [5] as compared to the ancestral strain of the virus. Therefore, there is a critical need to surveil and prepare for the genesis of novel variants while also learning from and responding to current VOC.

Although the currently observed SARS-CoV-2 VOCs might not represent true antigenic drift variants, as is observed periodically in the case of influenza viruses that re-infect a substantial portion of individuals with prior exposure to the original antigen [6,7,8], SARS-CoV-2 VOC do represent mutational paths for evolution of antigenic drift. Further, observation of convergent evolution of mutations on VOC in combination with studies of seasonal coronaviruses [9,10] suggests that mutations will accumulate across the spike protein in response to selection pressure from host antibody responses. Therefore, there is a need to develop tools to understand and potentially predict the evolving landscape of mutations with antigenic consequences in the receptor binding domain (RBD) and N-terminal domain (NTD) of the SARS-CoV-2 spike protein towards guiding vaccine development efforts and public health responses.

The RBD and NTD present numerous epitopes targeted by neutralizing antibodies that are overlapping and also diverse from the standpoint of the mode of engagement of the antibodies with these surfaces. RBD epitopes have thus far been described using a variety of approaches, including the classification of antibodies targeting the RBD into four major “classes” based on structural analysis [11]. Such discrete descriptions have proven useful for understanding key VOC mutations: the E484K mutation was associated with escape from class 2 antibodies while mutations at K417 were associated with escape from class 1 antibodies [12]. However, discrete descriptions do not convey heterogeneity within nor overlap between epitopes or antibodies. For example, it is not clear to what extent there remains class 1 and 2 antibody pressure on VOCs bearing the E484K and K417N/T mutations, especially from antibodies that do not fit neatly into these classes or that partially overlap with another class. Similarly, it is difficult to ascertain the degree to which combinations of mutations such as L452R + E484K might provide complementary escape with one another. Therefore, there is a need for alternative *continuous* descriptions of RBD antigenic space that capture epitope overlap and orthogonality from the stand point of the polyclonal antibody response and that distill these features down to the residue level for interpretability. Further, these descriptions should be extended to NTD given the significant role that NTD-directed antibodies play in the neutralizing antibody response [13]. Such an “antigenic space” description could guide prediction of the mutational landscapes that might result in clinically-relevant antigenic drift, particularly in a complementary manner on VOC with certain existing antigenic mutations.

In this study, we first seek to identify the spike protein sites under antigenic pressure based on computational and structural analyses of over 50 mAb/nanobody-RBD/NTD epitope-paratope interfaces. From this survey we obtain a map of RBD and NTD antigenic space that extends existing epitope definitions by describing epitope overlap and orthogonality at the residue level. We integrate the antigenic space map with an estimate of residue mutability derived from genetic, structural, and functional datasets to highlight potential antigenic drift sites (PADS) that appear both antigenic and mutable. 

Further, through examination of epitope overlap within our map we find that the VOC mutations with the largest individual escape effects (e.g., E484K and L452R) tend to occur within the overlap of multiple epitopes, suggesting that the escape potency of key variant mutations may be in part due to providing escape from many antibodies targeting overlapping epitopes centered around these sites. We identify NTD residues that play a similar multi-epitope function within the NTD supersite. Examining NTD multi-epitope mutations across variants, we find that the Gamma variant lacks such a mutation potentially explaining its relatively weaker antibody evasion than the mutationally homologous Beta variant which features NTD multi-epitope mutations. We further identify additional RBD multi-epitope escape sites such as R346 (observed to mutate on the Mu variant) within the overlap of RBD epitopes outside of the ACE2 RBS. 

Finally, through investigation of epitope orthogonality within our map we find that the escape strength of nine prominent variants is correlated with the degree to which the given variant’s mutational fingerprint covers the orthogonal compartments of RBD and NTD antigenic space. This finding suggests that variants with greater degrees of antibody escape leverage mutational complementarity (e.g., E484K + K417T) rather than accumulation of multiple functionally similar mutations (e.g., E484K + L452R) to efficiently escape from the polyclonal antibody response directed against spike. We integrate the findings from this study to present a framework for predicting additional mutations that may complementarily augment antibody evasion for the Beta and Delta variants—the most evasive and transmissible variants identified at the time of this investigation.

## 2. Materials and Methods

### 2.1. Epitope-Paratope Networking Calculations

For each protein or protein-complex, significant interaction network (SIN) scores between every pair of residues within the structure were computed as described previously [14]. Briefly, SIN computations consider all contacts between both side-chain and backbone atoms, and weight these contacts according to the energetics of the expected interaction. Interactions examined include hydrogen bonds, pi bonds, disulfide bonds, polar interactions, salt bridges, and Van der Waals interactions. Given that the SIN computation is weighted toward side chain interactions, glycine networking was interpolated from nearby residues to estimate the local effect of glycine mutations. Using the SIN output, the following metrics were defined and computed for all surveyed structures (Table S3; [11,15,16,17,18,19,20,21,22,23,24,25,26,27,28,29,30,31,32,33,34,35,36,37,38,39,40,41,42]). Within RBD/NTD Networking: The sum of all interactions between a given residue on RBD/NTD and all other residues on RBD/NTD. Direct Paratope Networking: For a given residue on RBD/NTD, the sum of all interactions between the RBD/NTD residue and all residues on the complexed antibody/nanobody. Indirect Paratope Networking: For a given residue on RBD/NTD, the sum of all interactions between the RBD/NTD residue and all other residues on RBD/NTD which are directly networked to an antibody/nanobody paratope. Total Paratope Networking: For a given residue on RBD/NTD, the sum of direct and indirect paratope networking scores. Note that for all networking measures, scores are normalized to the highest networking score within each RBD-mAb complex before they are subsequently compared.

### 2.2. RBD Mutations and Surface Complementarity Calculations

The impact of RBD SNPs on epitope-paratope surface complementarity and binding energy (dG) was modeled in PyRosetta [43] using the InterfaceAnalyzerMover. Absolute surface complementarity perturbations were scored relative to the wild type interface surface complementarity after repacking.

### 2.3. External Dataset Import and Processing

GISAID sequences were accessed and downloaded on 7 June 2021 [44]. Mutation frequencies at all RBD sites were computed for every observed mutation. For ACE2 Binding and RBD Expression raw data were accessed and downloaded from the Starr et al., supplemental file “mmc2.csv” on 19 January 2021 [45]. For residue-level computations mutation scores were averaged at each site for all SNPs. The set of RBD SNPs was computed from the SARS-CoV-2 reference genome (NCBI RefSeq NC_045512.2).

### 2.4. Epitope-Paratope Interaction Matrix and Clustering

mAb-epitope clustering: Direct, Indirect, and Total networking scores for each residue across all mAb and nanobody complexes were plotted using clustermap from the Seaborn statistical data visualization package with the ‘correlation’ distance metric and the ‘average’ linkage method [46]. Mutability Clustering: Mutability clustering of all RBD SNPs was performed using the computational and experimental features described in Table S1. GISAID mutation frequencies were log-transformed, all features were standardized, and then spectral clustering was performed on the affinity matrix generated according to pairwise Euclidean distance (sklearn SpectralClustering [47]). Clustering graphs were visualized using tSNE, and the accompanying cluster descriptions were computed as average scores for each feature for each cluster, except for the GISAID feature which was reported as the percentage of the cluster with an observed mutation in GISAID.

### 2.5. Antigenic Space Map

**Figure 1:**
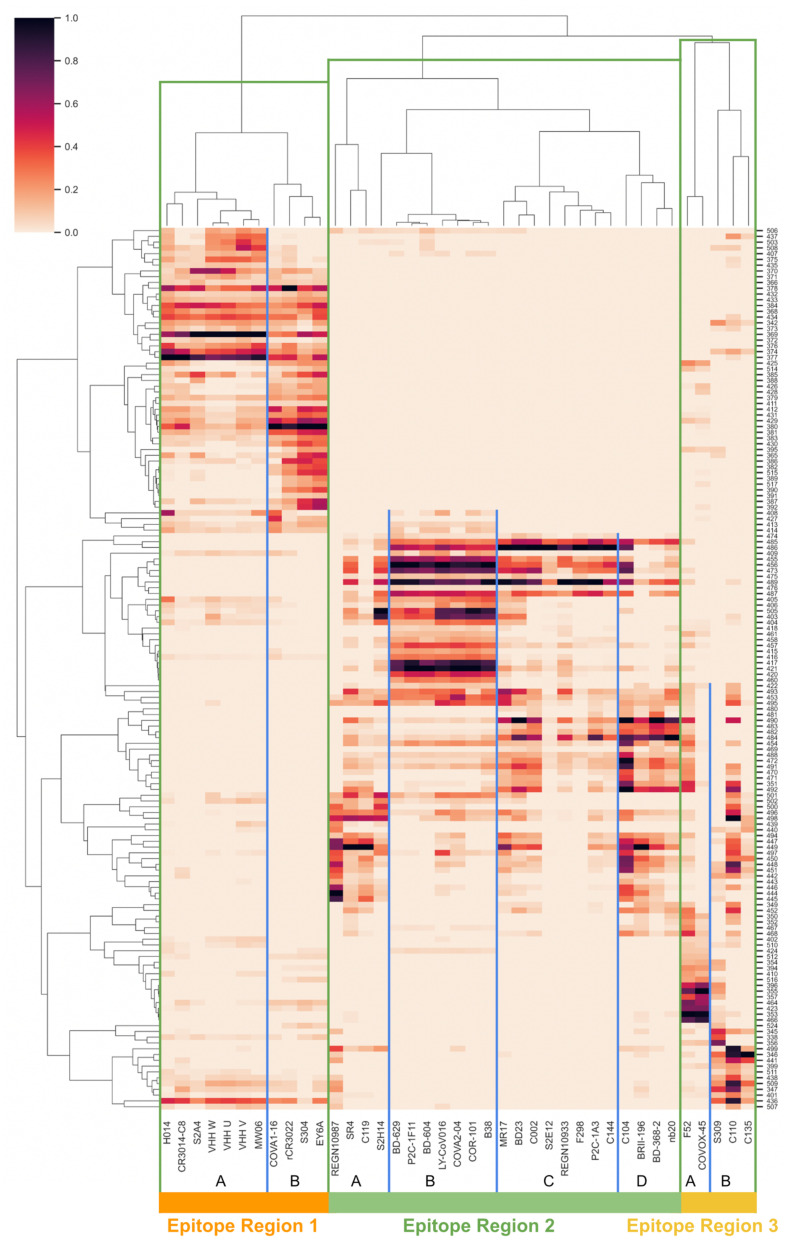
RBD Epitope-Paratope Interaction Matrix. The epitope-paratope interaction (EPI) matrix summarizes interactions between the set of Abs/nanobodies and all antigenic sites on RBD. The color of each square in the matrix represents total EPI networking between a given paratope (x-axis) and RBD site (y-axis). To aid visualization, only residues residues that score in the top 75% of EPI networking score for at least three of the mAbs or nanobodies are shown. Equivalent maps for NTD and for direct and indirect networking in isolation are included in the Supplement (Figures S1–S3). According to paratope clustering, three epitope regions (ERs 1–3) are identified and separated by vertical green lines. Within each ER, two to four epitopes are annotated (A–D) and separated by vertical blue lines, reflecting groups of Abs and nanobodies with tightly overlapping epitopes. The continuity of the EPI matrix highlights the heterogeneous nature of epitopes, displaying how epitopes overlap and providing a basis for computing antigenic orthogonality between RBD sites.

The Antigenic Space Map was constructed from a network model of RBD or NTD, in which nodes represent residues, node size is proportional to estimated antigenicity (RBD: Principal component analysis based on direct networking, indirect networking, surface complementarity perturbation, and binding energy perturbation (see Table S2); NTD: direct networking and indirect netowrking), node color is assigned to mutability and relative antigenicity rankings (e.g., PADS-30 → top 30% of most antigenic PADS), and relative node positions represent functional orthogonality in antigenic space. Node positions were computed from edge weights via the Fruchterman-Reingold force-directed algorithm. Edge weights were calculated according to cosine similarity between residue-pairs in the epitope-paratope survey (Figure 1) and implemented via the sklearn pairwise_distances function [47]. Edges are only draw between nodes with cosine similarity greater than 0.5.

## 3. Results

### 3.1. RBD and NTD Epitope-Paratope Interface Survey

Toward continuously describing RBD and NTD antigenic space, we first surveyed over 50 RBD and NTD epitope-paratope interfaces by extending a previously validated computational approach for quantifying protein networks, known as Significant Interaction Networks [14,48]. From the network model of epitope-paratope complexes, we derived networking scores between each RBD or NTD epitope residue and each antibody paratope (see Methods), resulting in a matrix describing the interactions between all RBD/NTD sites and all antibodies surveyed (RBD: Figure 1; NTD: Figure S2). The RBD and NTD matrices were further clustered epitope-wise (RBD/NTD residues) and paratope-wise (antibodies). For RBD, the mAb clustering (Figure 1, X-axis dendrogram) indicates three “epitopes regions” (ERs) on RBD, where an epitope region is defined as a set of overlapping antibody epitopes (overlapping epitopes labeled A–D for each ER). The three RBD ERs broadly correspond to the RBD back (ER-1), ACE-2 binding site (ER-2), and RBD front (ER-3). For NTD, the antibodies cluster into a single dominant epitope region that is consistent with previous descriptions of the NTD “supersite” [38] and a second under-sampled group that is best annotated as the DH1052 antibody epitope [42].

Importantly, the epitope-paratope interface matrices describe polyclonal epitopes continuously in relation to one another at the residue level, thus enabling characterization of epitope overlaps and computation of orthogonality between epitopes. To further investigate the relationships between overlapping epitopes and to present an interpretable framework for understanding these relationships, we next derived an antigenic space map of RBD and NTD from the epitope-paratope interface matrices. For RBD, we first focus on RBD ER-2 (ACE2-binding site epitopes) given its immunodominance, potent neutralization when targeted, and that key VOC mutations K417N/T, L452R, E484K, and N501Y occur in this ER.

### 3.2. RBD and NTD Antigenic Space Map

**Figure 2:**
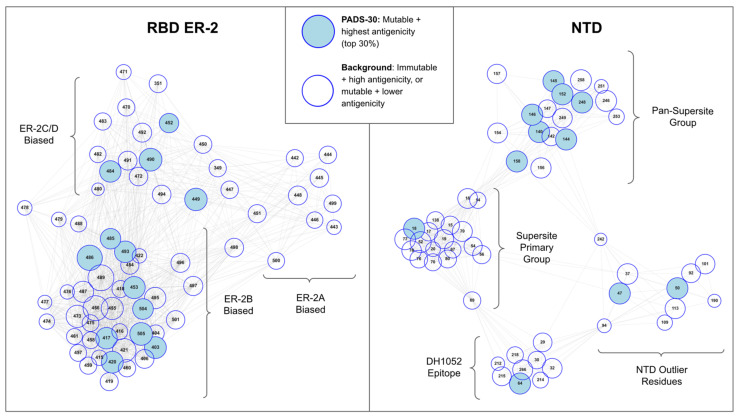
Map of RBD ER-2 and NTD Antigenic Space. RBD or NTD residues are represented as nodes with node size proportional to estimated antigenicity and node color representative of mutability and antigenicity ranking (PADS-30 designation). Node positions and edges are determined based on cosine similarity between sites in the epitope-paratope interaction matrices (Figure 1 and Figure S1) such that clusters of nodes describe groups of residues playing similar functions as epitope constituents (interacting with similar sets of antibodies with similar relative strengths). Residue clusters are assigned descriptive names according to orientations toward a specific epitope region from Figure 1 (e.g., ER-2B biased). Key RBD escape sites E484 and L452 cluster together (ER-2C/D biased) suggesting that antibodies targeting ER-2C/D play a critical role in the neutralizing response directed against the RBD RBS. Further, certain RBD residues (E484, G485, F486, and Q493) reside at the edge of one ER-2 cluster and adjacent to another cluster due to these sites interacting with antibodies targeting both clusters. This effect occurs as a result of these sites occurring within epitope overlap regions suggesting that these sites play a multi-epitope role within ER-2. Similarly, a cluster of NTD sites is identified to play an analogous “pan”-epitope region role by interacting with nearly all Abs that target the NTD supersite. The antigenic space map is thus a framework for visualizing key antigenic sites and their features (antigenicity, mutability, and functional orthogonality mapped to Euclidean distance).

From the RBD and NTD epitope-paratope matrices, we derived an antigenic space map that illustrates the relationships between residues as functional components of the polyclonal antibody response. That is, we developed an antigenic space map that seeks to present (1) residue antigenicity, (2) residue mutability, and (3) residue assignment to a component of the antibody response directed against RBD or NTD (Figure 2). We briefly describe the construction of this map, highlighting the key features, and then subsequently apply the map to analyze VOC mutations, relative VOC escape strength, and predict potential future antigenic drift paths.

The antigenic space map was constructed as follows. First, we assigned residue antigenicity in our map to node size, where antigenicity is defined as the importance of a given residue as an epitope constituent across the antibodies surveyed. Residue antigenicity was estimated using a principal component analysis describing epitope-paratope networking and epitope-paratope surface complementarity perturbation upon mutation (see Methods). Second, we estimated residue mutability according to genetic, structural, and functional constraints on mutation of a given residue (see Methods). The residues scoring highest in antigenicity and mutability are denoted potential antigenic drift sites (PADS) and identified via node color. Third, we mapped each residue’s function as an epitope constituent in the immune response directed against RBD/NTD by computing the orthogonality between residues in the epitope-paratope interface matrix via cosine similarity. This process assigned functionally similar residues to similar locations in our antigenic space map. That is, a pair of RBD residues interacting with an identical subset of antibodies (and with similar relative strength across Abs) in the epitope-paratope interface matrix would be placed in overlapping positions within the antigenic space map. In contrast, RBD residues interacting with inverse subsets of antibodies serve orthogonal functions as epitope constituents across the polyclonal response directed against RBD and would therefore occupy highly distant positions within the antigenic space map. The antigenic space map thus concisely presents residue antigenicity, mutability, and relationships between epitope residues as overlapping or orthogonal functional epitope constituents.

### 3.3. Residue Clusters within the Antigenic Space Map Describe Residue Function

Examining the antigenic space map of RBD ER-2 we find three clusters of residues which suggests three functional groups describe the antigenic space of the ACE2-binding site epitopes (ER-2). Cross-referencing these residue clusters with the roles of the residues in epitope-paratope interfaces (Figure 1), we observe that the first cluster consists of sites most strongly interacting with antibodies targeting ER-2C and ER-2D—the “ER-2C/D biased” cluster. We find that the VOC mutations E484K and L452R measured to have the greatest individual effect on escape from neutralization [49] reside within this cluster. Further, variants have been detected that appear best defined by mutations at these ER-2C/D biased sites [50,51] including at sites other than E484 and L452 such as E471 [52]. As this cluster contains the most impactful VOC escape mutations for antibody escape, these data suggest that antibodies targeting ER-2C/D may currently exert the most antigenic pressure on spike evolution. Interestingly, this group is also the only ER-2 cluster that spans two epitopes (C and D), suggesting that mutations at sites within this cluster may also be particularly impactful due to occurring in a region of epitope overlap.

Next, we find a second ER-2 cluster best described as “ER-2B biased” as the sites in this cluster interact most strongly with antibodies assigned to ER-2B in Figure 1 such as LY-CoV016 and IGHV3–53 germline antibodies [53,54]. VOC mutations with small to moderate individual effects on antibody evasion such as K417N/T reside within this cluster. Additionally, this cluster is ovoid shaped with certain residues in the northern portion (G485, F486, Q493) positioned proximal to the ER-2C/D cluster. Similarly, E484 (within ER-2C/D cluster) is positioned adjacent to the ER-2B biased cluster. Examining these specific residues on cluster edges and considering the algorithmic basis for generation of the antigenic space map, we find that residues residing on the edge of one cluster and in close proximity to an adjacent cluster possess antigenic functions associated with both clusters. That is, of all of the sites in the ER-2B biased cluster, G485, F486, and Q493 tend to interact most strongly with antibodies assigned to ER-2C/D. Similarly, E484 interacts with antibodies targeting ER-2B more strongly than other residues in the ER-2C/D-biased group such as L452, perhaps explaining E484’s relatively stronger individual escape effect and clearer association with VOC with the strongest escape such as Beta and Gamma than L452. Thus, E484 and adjacent sites spanning these two clusters exhibit a multi-epitope function, interacting either directly or indirectly with nearly all antibodies that target the potently neutralizing RBD epitope region 2.

Third, we find a final ER-2 cluster termed “ER-2A biased” consisting largely of residues on the 443–450 loop whose unique location in antigenic space is imparted due to their interaction with antibodies targeting both ER-2 and ER-3 such as REGN-10987. Sites on this loop are estimated to be relatively less antigenic and less mutable than sites within the other two ER-2 clusters. As compared to the other two clusters, the ER-2A biased cluster is relatively low density, with certain residues such as Y449 and N450 spanning this cluster and the ER-2C/D cluster, suggesting that these sites reside within epitope overlap regions and may exhibit certain multi-epitope qualities.

Examining the NTD antigenic space map (Figure 2, right) we find two large clusters of residues representing two functional components of the NTD “supersite” and two smaller clusters representing the DH1052 epitope and outlying antigenic sites. Most significantly, we find that the two NTD supersite clusters do not bifurcate into sites biased toward separate epitopes or angles of attack as observed for RBD-ER-2. Rather, the two supersite clusters are distinct due sites in one cluster possessing a pan-epitope property while sites in the other cluster do not. That is, the residues of the Pan-Supersite Group tend to interact (directly or indirectly) with nearly every antibody that engages the NTD supersite, regardless of the antibody orientation or angle of attack, while the Supersite Primary Group residues interact with only a subset of the antibodies targeting the NTD supersite. Such a pan-epitope effect is supported experimentally for certain residues within the Pan-Supersite cluster such as W152, for which the W152C mutation was shown to escape binding from every NTD-directed tested [55]. Meanwhile, the DH1052 Epitope residues and NTD Outlier residues interact specifically with a single or a narrow subset of antibodies targeting NTD either at non-supersite epitopes (DH1052 Epitope) or due to a highly unique angle of attack of the supersite (Outlier Residue cluster). Interestingly, we find that two residues commonly mutated on VOCs (H69, L242) occupy positions in NTD antigenic space that span multiple clusters suggesting multi-epitope functions.

In addition to observing that well-known VOC mutations with significant contributions to antibody evasion are located in multi-epitope regions of antigenic space describing epitope overlaps (Alpha NTD: Y144, Beta/Gamma RBD: E484, Beta NTD: R246, Delta NTD: R158), we also note that other key VOC mutations stand out within our computational antigenicity and mutability estimates. In particular, K417 is identified as mutable, has a high estimated antigenicity, and occupies a central position within the ER-2B-biased cluster. Further, K417 is closely associated with residues that have been experimentally measured to have substantial escape effects but that are estimated as immutable due to associated fitness cost such as F456 [56]. Thus, K417 may convergently mutate due it being the most antigenic and mutable residue in this important region of ER-2 antigenic space, as nearby residues that may be more antigenic such as F456 are mutationally constrained. 

Further, we observe multi-epitope roles for P384 and I434 in RBD ER-1 and R346 in RBD ER-3 (Figure S6). R346 is of particular interest due to the occurrence of R346K on the Mu variant (formerly B.1.621) which is significant due to the Mu variant’s substantial escape from neutralizing antibodies [57]. Other VOC mutations identified as mutable and high antigenicity in the PADS-30 set include: F490 and Y248 (Lambda), L18 (Gamma), Y144 (Alpha), and R158 (Delta). Notably, N501 and T478 do not score sufficiently high on estimated antigenicity to be included in PADS-30; a finding that is consistent with N501Y and T478K mutations being selected primarily due to their enhanced effect on ACE2 binding [58] rather than for providing a substantial antibody evasion effect. Next, we sought to interrogate whether the constellation of mutations on a given variant might explain escape strength by examining the orthogonality feature of the antigenic space map.

### 3.4. VOC Escape Strength Is Explained by Antigenic Space Coverage

**Figure 3:**
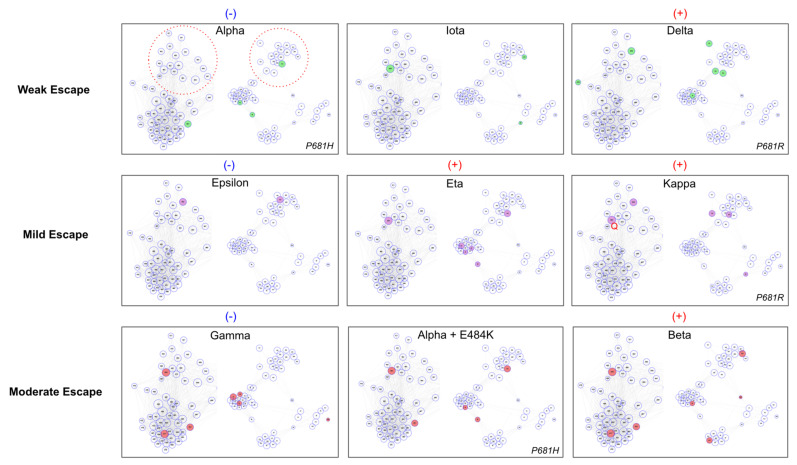
Nine Variants in RBD-ER-2 + NTD Epitope-Paratope Space. Variants with characterized in vitro escape via live virus neutralization [49] are projected in the antigenic space map from Figure 2 according to their relative reduction in PRNT50. The VOCs are divided into three groups for weak, mild, and moderate escape and further assigned blue (−) or red (+) to indicate relative escape magnitude within each horizontal grouping. The RBD ER-2C/D and NTD pan-supersite clusters are highlighted for reference as red dotted circles in the Alpha variant panel due to key escape residues occurring in these locations. The ER-2A biased group is cropped for clarity as none of the included variants feature mutations in this cluster. Variants with weak or mild escape tend to have a single ER-2C/D biased escape mutation (E484K or L452R) in combination with poor coverage of NTD clusters. Variants with mild escape tend to have relatively stronger NTD coverage (e.g., Eta vs. Iota) than variants with weak escape. VOCs with moderate escape feature mutations in two ER-2 clusters (e.g., E484K in ER-2C/D + K417N in ER-2B), and their relative escape strength is correlated with the degree of mutational coverage of the NTD clusters (i.e., Beta mutations span all four NTD clusters while Gamma’s NTD mutations occur within the same NTD cluster suggesting functional redundancy). Further, Gamma is the only variant that lacks a pan-supersite NTD mutation, potentially explaining its relatively weaker antibody evasion than Beta.

Toward understanding how constellations of mutation might explain variant antibody evasion, we annotated the ER-2 + NTD antigenic space map with the mutations of nine major variants whose escape from neutralization had been previously measured experimentally using live virus [49] (Figure 3). We order the nine variants according to fold-reduction in neutralizing titer measured in Lucas et al., 2021 which ranges from weak escape (Alpha: < 2-fold reduction) to moderate escape (Beta: ~6-fold reduction). 

We find that variants with weak escape tend to have little coverage of antigenic space while variants with stronger escape feature constellations of mutations with comprehensive coverage of ER-2 + NTD antigenic space. Specifically, we find that variants with weak or mild escape tend to have a single ER-2C/D biased mutation (E484K or L452R) and poor coverage of NTD antigenic space (a single NTD mutation or NTD mutations concentrated within the same NTD cluster). Relative scoring within the weak/mild group is logically explained by antigenic space coverage: Alpha measures the weakest escape and lacks an ER-2C/D biased mutation; Eta/Kappa measure the strongest escape within the weak/mild group and feature at least one ER-2C/D biased mutations in combination with mutations in multiple NTD clusters. The Delta variant is an outlier to this trend with relatively greater mutational coverage than other weakly evading variants, however, we note that Delta features the P681R mutation that has been suggested to enhance susceptibility of live virus to antibody neutralization [49]. Meanwhile, variants in the moderate escape group (Gamma, Alpha + E484K, and Beta) feature both ER-2C/D biased and ER-2B biased mutations combined with moderate to strong coverage of NTD antigenic space, with the relative escape strength of these variants correlated with their degree of coverage of NTD clusters.

The antigenic space framework provides mechanistic explanations for phenotypic differences between variants. A key open question on relative escape between variants asks why Beta measures stronger antibody evasion than Gamma despite nearly identical RBD mutations and numerous NTD supersite mutations. The antigenic space map illustrates how the constellation of Beta NTD mutations spans the various orthogonal compartments of NTD antigenic space suggesting that the Beta NTD mutations are complementary to each other, while the Gamma NTD mutations are tightly concentrated in a single cluster (Supersite Primary Group) suggesting that the Gamma NTD mutations are functionally redundant with one another in terms of antibody escape. Additionally, Gamma is surprisingly the only variant of the nine examined that lacks a pan-supersite mutation that putatively affects binding of all antibodies targeting the NTD supersite.

### 3.5. Sites of Concern for the Delta and Beta Variants

**Figure 4:**
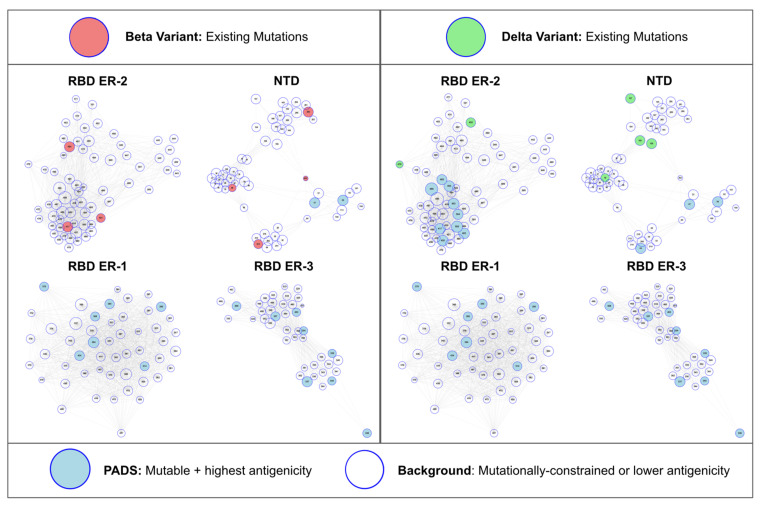
Beta and Delta Variants in RBD + NTD Antigenic Space. Current Beta and Delta variant mutations are illustrated in antigenic space as red or green highlights, respectively. In regions of antigenic space that are not currently mutated for these variants, we annotate the highest mutability and antigenicity sites (PADS) in these unmutated regions as sites of potential complementary antibody evasion upon mutation. That is, mutations in these regions are most likely to knockdown *orthogonal* components of the antibody response directed against RBD and NTD alongside existing Beta and Delta variant mutations. Specifically, the Delta variant lacks mutations in the RBD ER-2B, NTD DH1052, and NTD outlier clusters. The Beta variant mutations have largely covered all ER-2 and NTD clusters. Neither variant currently features mutations in RBD ER-1 or RBD ER-3, suggesting escape complementarity for mutations in these regions (Figure S5).

Given the association between antigenic space coverage and stronger escape from neutralization we sought to present a framework for understanding which mutations on VOC may be more likely to result in complementary escape alongside existing mutations on these VOC. From the set of potential antigenic drift sites (PADS) that we previously estimated to be both mutable and antigenic, we identify a refined set of high-risk sites for the Beta and Delta variants (Figure 4). From the map of ER-2 + NTD antigenic space, we observe that the Delta variant lacks ER-2B biased and NTD-DH1052 epitope mutations. While K417N/T are the most prolific ER-2B mutations that could provide this effect, the PADS-30 set suggests that mutations at sites 403, 420, 453, 485, 486, 493, 504, and 505 might provide a similarly complementary effect alongside existing Delta mutations in terms of knocking down orthogonal components of the polyclonal antibody response directed against Delta RBD. Still, K417N has already been identified on sub-lineages of Delta and we therefore advise careful surveillance of such sub-lineages as we expect them to benefit from enhanced escape relative to Delta. Within NTD, we predict mutations at sites 64, 69, 95, and 215 to be most complementary alongside the existing Delta mutations. Further, while we have thus far only presented an RBD map of ER-2 antigenic space as a main figure in this manuscript, we now note that based on our initial epitope-paratope survey (Figure 1) RBD bears two additional epitope regions (ER-1 and ER-3) both of which have not mutated for any VOC identified thus far. Mutations in ER-1 and ER-3 would thus be logically complementary alongside the existing mutations on all VOC in terms of knocking out an orthogonal component of the antibody response directed against RBD.

While ER-2 is immunodominant and the most potent target of neutralizing antibodies [59], the polyclonal antibody response to infection and vaccination still targets other regions of RBD described by ER-1 and ER-3 (Figure S5). Therefore, as SARS-CoV-2 begins to face increasing antigenic pressure from populations with majorities of convalescent or vaccinated individuals, it is possible that VOC may increasingly benefit from mutations at these less immunodominant sites. We present an antigenic map that features all epitope regions of RBD in addition to NTD (full maps of ER-1 and ER-3 are provided in Figure S5), and highlight all PADS sites in ER-1 and ER-3 that may be most likely to mutate under antigenic pressure on the Delta and Beta variants. In particular, we identify certain multi- and pan-epitope sites within these ERs that are involved in epitope-paratope interactions for the majority of antibodies targeting these epitopes analogous to ER-2 sites such as E484 and L452. Learning from the convergent evolution of multi-epitope ER-2 mutations E484 and L452, we anticipate that ER-1 and ER-3 multi-epitope sites such as R346 and P384 pose a risk as similar mutation hotspots on future variants.

## 4. Discussion

In this study, we integrated an epitope-paratope interface survey of over 50 RBD/NTD-Ab/nanobody complexes along with experimental datasets describing genetic, structural, and functional constraints on mutation to identify RBD and NTD residues that appear the most mutable and highly targeted by antibodies—the set of potential antigenic drift sites (PADS). The epitope-paratope survey presents a continuous representation of RBD and NTD epitope regions that highlights how antibodies target overlapping or divergent epitopes across RBD and NTD. Further, we find that antibody clustering within the epitope paratope interface matrices captures certain antibody functional qualities, for example distinguishing antibodies that neutralize via avidity [27] (antibodies in ER-1A) from antibodies that neutralize via ACE2 binding interference [23] (antibodies in ER-1B).

Next, we developed an RBD + NTD antigenic space map: a novel framework for describing polyclonal RBD and NTD epitopes with a particular focus on epitope overlap and orthogonality. This framework allows us to identify key residues within RBD and NTD epitope regions that play a multi- or pan-epitope function by interacting with large subsets of antibodies targeting a given epitope region. Further, we apply the framework to nine variants, finding that variants with relatively stronger antibody tend to have greater coverage of the various orthogonal components of RBD and NTD antigenic space. That is, variants with stronger escape possess mutations that cover the epitope regions of the polyclonal antibody response directed against these domains, while variants with relatively weaker escape tend to accumulate mutations within the same or overlapping epitope regions. Finally, we leveraged the antigenic space framework to predict which residues pose the greatest risk of mutating with complementary effects on the degree of antibody evasion for the Beta and Delta variants.

Our findings provide mechanistic explanations for open questions regarding VOC antibody evasion. Foremost, we present an explanation for the mechanism by which the Beta variant has greater antibody escape than the Gamma variant despite these two VOC sharing nearly identical RBD mutations and structurally similar NTD mutations. Our antigenic space framework suggests that the NTD supersite features certain supersite residues that interact with nearly all antibodies targeting the supersite (the Pan-Supersite Group), while other supersite residues only interact with a subset of supersite-directed antibodies. Indeed, we observe that the Gamma variant is the sole variant of the nine variants investigated that lacks a pan-supersite mutation, and propose this distinction from the Beta variant as an explanation for Gamma’s relatively weaker escape from antibodies.

Though we do not observe analogous sites within RBD that interact with all antibodies targeting this domain, we find that the RBD ER-2 residues with the largest individual escape effect (E484 and L452) occupy a multi-epitope cluster (ER-2C/D biased) that interacts with a large subset of the antibodies targeting ER-2. Further, E484 is located proximal to the ER-2B biased cluster suggesting that E484 interacts with additional antibodies targeting ER-2B in addition to antibodies targeting ER-2C/D. This finding is supported by examination of the RBD epitope-paratope matrix (Figure 1) in which E484 is networked to nearly all ER-2 antibodies. These observations suggest that E484 may uniquely play a multi-epitope role across RBD ER-2, offering a hypothesis for E484’s convergent evolution across multiple VOC. Notably, such convergent evolution is not observed within NTD, which is logical in the context of our findings as a number of NTD supersite residues exhibit a pan-supersite function suggesting multiple functionally similar solutions. That is, many NTD sites appear capable of producing pan-supersite escape upon mutation, while the number of such multi-epitope sites on RBD may be restricted to only a small number of mutable residues such as E484 and L452.

For both RBD and NTD, our study supports the conclusion that residues residing within epitope intersections may be hotspots possessing pan-Ab escape effects. We highlight a number of residues across other RBD and NTD epitopes that may be similar escape hotspots to E484. In particular, we highlight R346 which we find to uniquely serve a pan-epitope function for RBD ER-3 (RBD front; Figure S6). These results suggest that the R346K mutation on the Mu variant may contribute to Mu’s substantial antibody evasion. If R346K is shown experimentally to contribute to Mu’s antibody evasion, our manuscript suggests a mechanistic basis for such evasion, with R346 mutations playing an analogous functional role to E484 mutations from the perspective of multi-antibody escape.

While the antigenic space map serves as a framework for understanding where future mutations may occur on existing variants within ER-2 and NTD, we note that none of the nine VOC examined feature mutations in either RBD ER-1 or ER-3. Given that these epitopes are less immunodominant, less accessible or inaccessible in the RBD down conformation, and that that a portion of antibodies targeting these epitopes are weak neutralizers or non-neutralizers [37], it is plausible that these ERs have not experienced significant antigenic pressure through the pandemic to date. Still, potently neutralizing antibodies targeting these epitope regions have been isolated from the sera of convalescent individuals (e.g., EY1A [33] and S309 [60]). Further, SARS-CoV-2 antibodies that fail to neutralize in vitro can still contribute to in vivo protection as evidenced by DH1052 [42]. A number of the PADS in these ERs such as P337, E340, and R346 are surface exposed and have been documented as escape mutants from individual mAbs [61,62]. Antigenic mutations in these epitope regions could therefore still be of consequence for variant evasion, pathogenicity, and mAb therapeutics.

VOCs with mutations in RBD ER-1 and RBD ER-3 may emerge in the future course of the pandemic if certain conditions are met [63]. Conditions promoting antigenic pressure on these sites may include (1) a critical level of a local population has been infected or vaccinated such that mutations resulting in antibody evasion provide a substantial fitness advantage to variants, and (2) antibodies targeting the dominant ACE2 RBS and NTD epitopes have already been sufficiently evaded by mutations in ER-2/NTD on circulating variants such that mutations in the less immunodominant ER-1 and ER-3 epitope regions offer a fitness advantage. Alternatively, novel epitopes formed by mutations in the RBS and NTD epitopes may still prove more immunologically relevant than ER-1/3 epitopes with relatively lower neutralizing potential, such that antibodies targeting the mutated ACE2 epitopes continue to dominate the sera response and exert stronger antigenic pressure on ER-2 than on ER-1/3. Still, the immunodominance landscape is complex and dynamic, and mutations such as P681R may indirectly affect antigenic pressure on ER-1/3 [49]. Whether or not variants beyond Mu (with R346K) develop mutations within ER-1 and ER-3 remains to be seen, but the potential for mutations within these regions that are complementary with existing VOC mutations presents a potential antigenic drift risk and highlights the need for continued global variant surveillance and characterization.

## 5. Conclusions

We conclude by highlighting that so far in the SARS-CoV-2 pandemic immune escape has not been the primary driver of variant success. This fact is evidenced by observing that the VOCs with the strongest escape effects (Beta and Gamma, [64,65]) were outcompeted by the Alpha and Delta variants featuring relatively weaker escape from neutralizing antibodies [49,66,67]. The dominance of Delta over Beta and Gamma as a result of replication advantages due in part to increased fusogenicity [68] is logical given that the majority of the world’s population are not vaccinated nor convalescent. Even in countries with unrestricted access to vaccination substantial numbers of naïve individuals remain. It is therefore understood that selection on the basis of replication and transmission within naïve populations has dominated variant dynamics for the majority of the pandemic thus far [69]. However, this fitness equation may be in the process of changing—or have already changed—as the Delta variant encounters communities with substantial existing antibody protection [70]. Indeed, the recent observation of a “Delta Plus” variant bearing the K417N mutation [71] is consistent with the predictions outlined in this manuscript based on PADS and the Antigenic Space Map in which ER-2B-biased mutations including K417N are highlighted as the most likely path for the Delta variant to non-redundantly mutate toward increased antibody evasion.

While predicting VOC emergence and phenotype governed by replication and transmission advantages is difficult or impossible to model and predict, the pandemic’s next phase may be governed by antigenic pressures that are already well-characterized as described in this manuscript. The Antigenic Map framework presented here integrates the wide-ranging data required to chart and interpret the various orthogonal components of RBD and NTD antigenic space. We therefore hope our framework serves as a tool to understand the evolving landscape of antigenic mutations in the RBD and NTD of the spike protein towards guiding vaccine development efforts and public health responses.

## Data Availability

All structures analyzed are available from the Protein Data Bank (https://www.rcsb.org) according to the accession codes in Table S3.

## Supplementary Materials

The following Supplementary Materials are available online at https://www.mdpi.com/article/10.3390/v13102009/s1, Figure S1: Epitope-Paratope Matrix for N-Terminal Domain, Figure S2: Direct Epitope-Paratope Matrix for RBD, Figure S3: Indirect Epitope-Paratope Matrix for RBD, Figure S4: Mutability Clustering for RBD SNPs, Figure S5: PADS-40 for RBD ER-1 and ER-3, Table S1: RBD Spectral Clustering Features, Table S2: RBD Antigenicity PCA Features, Table S3: mAb/Nanobody—RBD/NTD Complexes.
