# Supplementary material for: An Antigenic Space Framework for Understanding Antibody Escape of SARS-CoV-2 Variants
Source: Viruses. 2021 Oct 6;13(10):2009. doi: 10.3390/v13102009 (PMC8537311; doi:10.3390/v13102009)
Supplement: Supplementary file 1 [file viruses-13-02009-s001.zip › viruses-1375181supplementary.pdf]

Supplemental Figures (S1-S7) and Tables (S1 to S3)

Figure S1. Total Epitope-Paratope Matrix for N-Terminal Domain

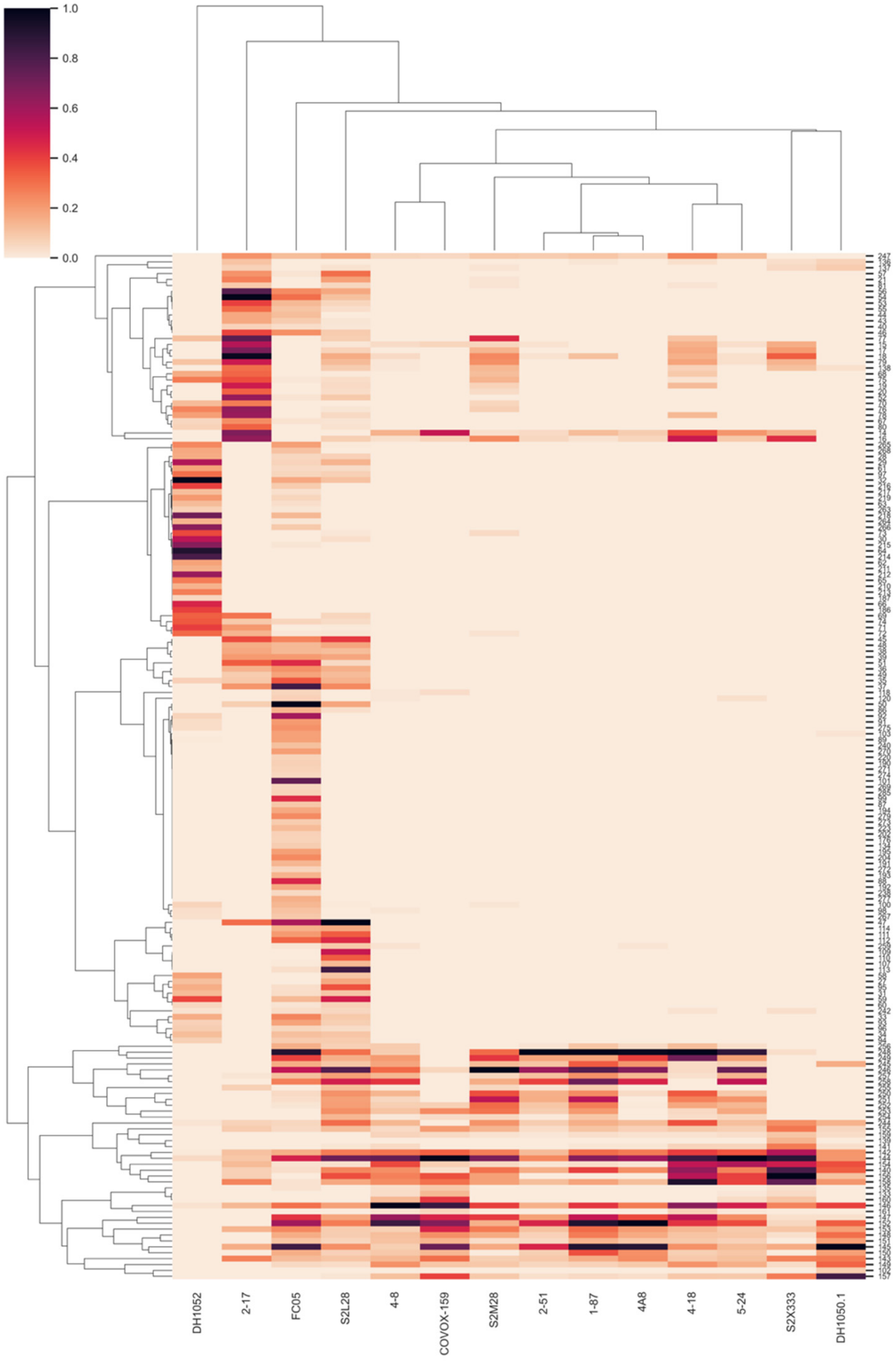

Figure S2: Direct Epitope-Paratope Matrix for RBD

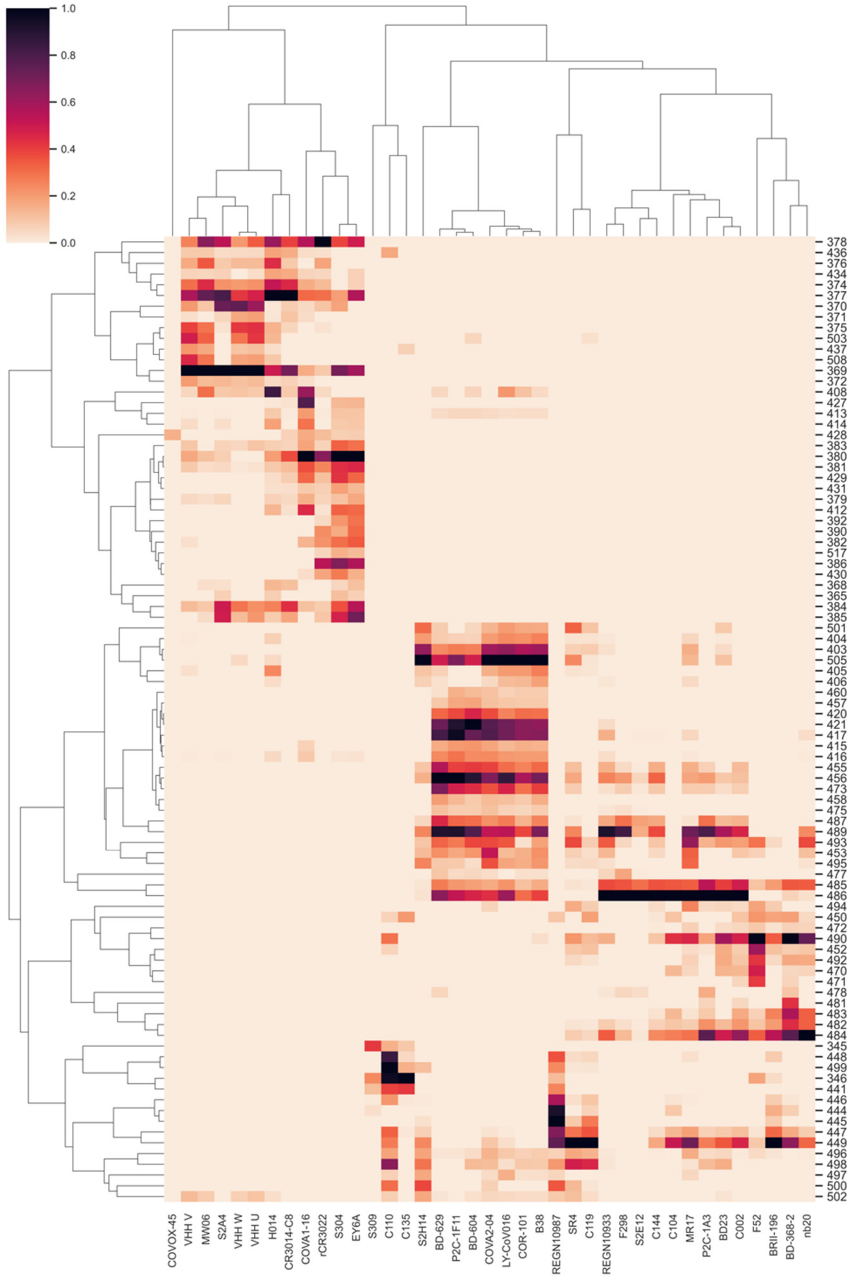

Figure S3: Indirect Epitope-Paratope Matrix for RBD

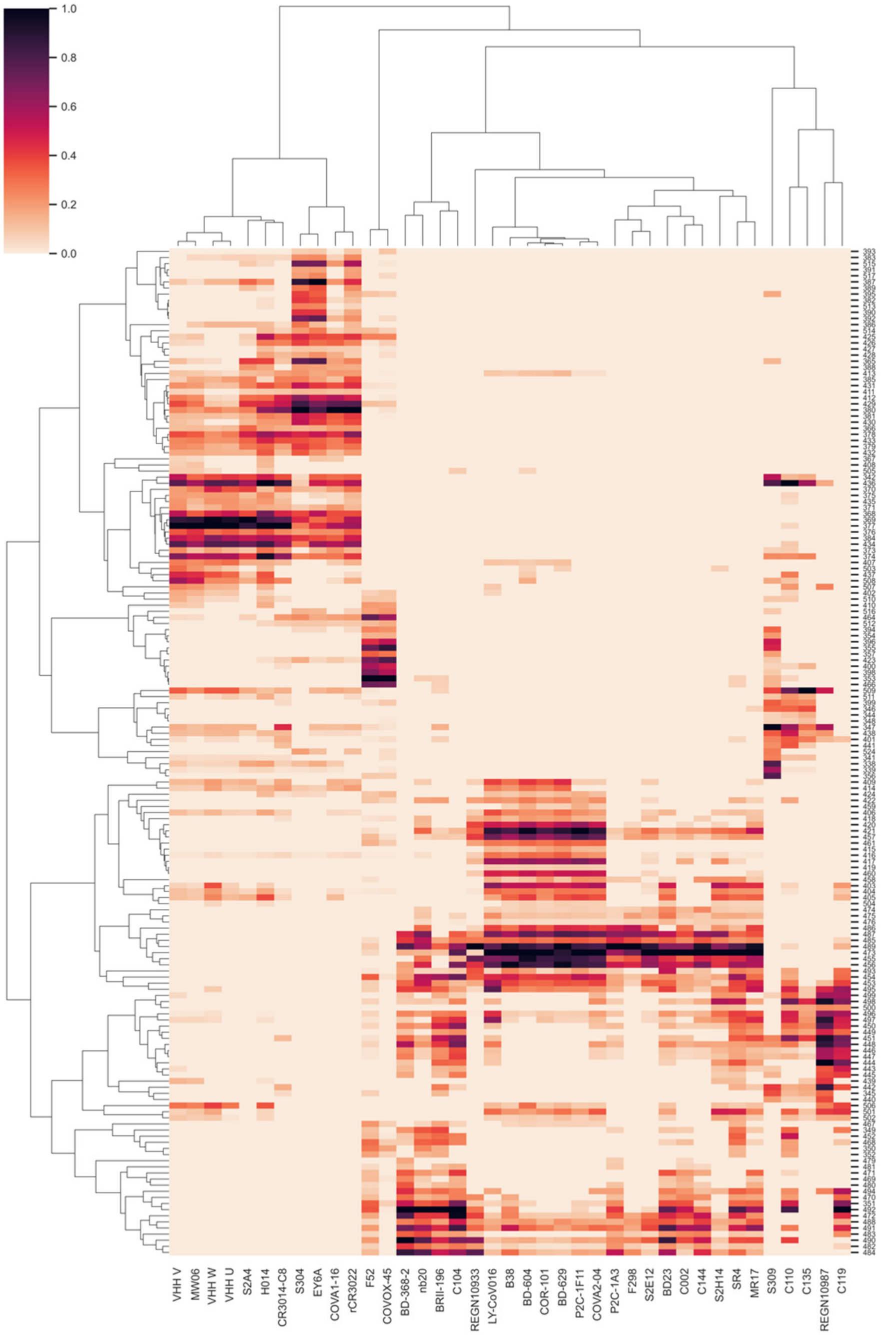

Figure S4: Mutability Clustering for RBD SNPs. Top, tSNE visualization of the clustering. Bottom, averaged and normalized values for each cluster along the feature set. Negative values imply knockdowns. Cluster 1 includes the RBD residues that appear the least genetically, structurally, and functionally constrained to mutate.

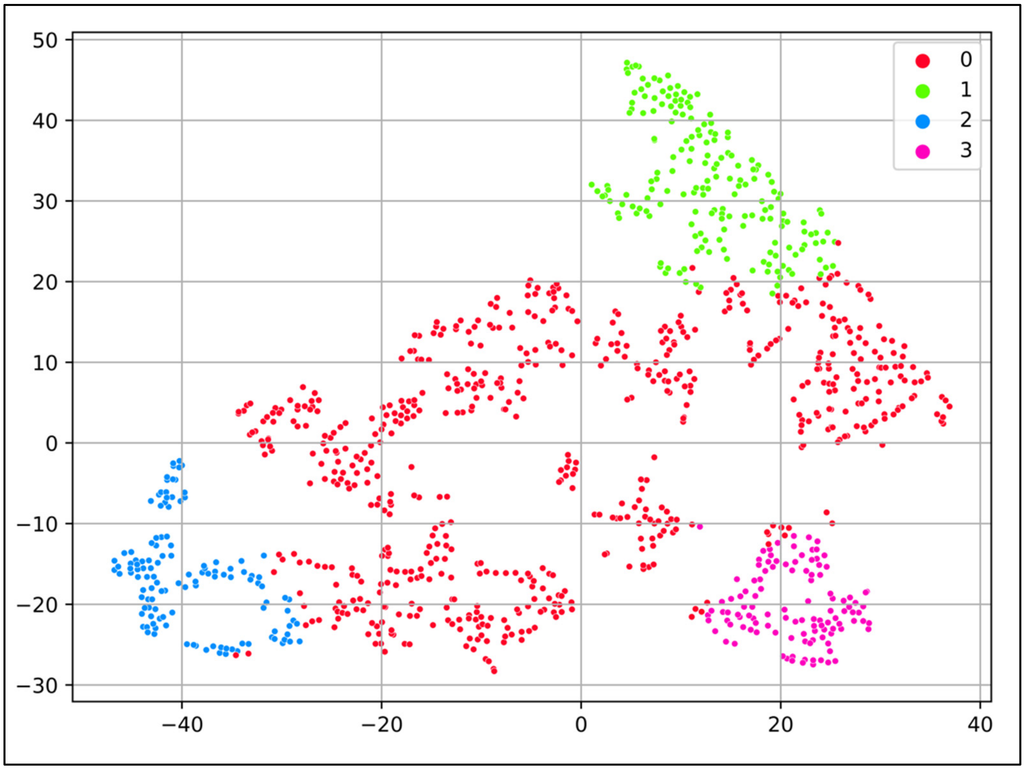

Cluster	RBD Expression	ACE2 Binding	Within RBD Networking	GISAID % Observed
**0**	-0.27	-0.13	0.32	52
**1**	-0.04	-0.02	0.16	100
**2**	-0.63	-0.83	0.40	5
**3**	-0.06	-0.02	0.12	0

Figure S5: PADS-40 for RBD ER-1 and ER-3. ER-1 features substantial similarity across all Abs targeting it, including CR3002, S304, and EY6A, and as a result we observe a single large cluster of residues. Pan-ER1 residues that interact most strongly with the largest subset of ER-1 mAbs reside in the center of the cluster, such as I368, K378, P384, and I434. Meanwhile, ER-2 separates into two clusters and the unique residue R346. ER-2 features little overlap across Abs, with one cluster containing S309-oriented sites and the second containing COVOX-45/F52 oriented sites.

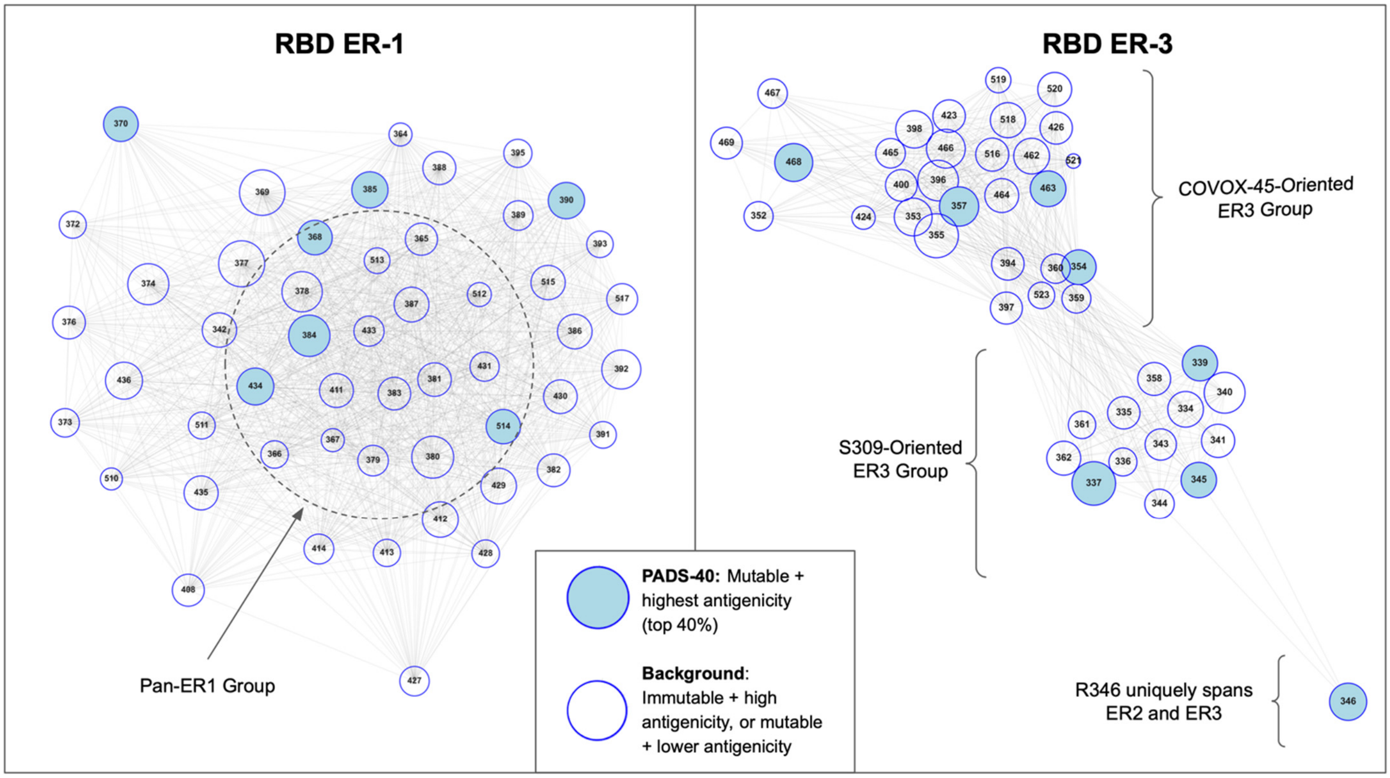

Table S1: RBD Spectral Clustering Features

	Genetic Feature	Structural Feature	Functional Feature
**Computational Tool**	SNP mask	Within RBD Networking (SIN)	N/A
**Experimental Dataset**	GISAID Mutation Frequencies [Shu et al., 2017]	RBD Δ Expression [Starr et al., 2020]	ACE2 Δ Binding [Starr et al., 2020]

Table S2: RBD Antigenicity PCA Features

PCA Feature	Tool
RBD-mAb Surface Complementarity Absolute Perturbation	PyRosetta – InterfaceAnalyzerMover
RBD-mAb Binding Energy (dG)	PyRosetta – InterfaceAnalyzerMover
RBD-mAb Direct Networking	EPCN - Direct
RBD-mAb Indirect Networking	EPCN - Indirect

Table S3: mAb/Nanobody – RBD/NTD Complexes

Antibody/Nanobody	PDB	Reference
**ACE2**		
N/A – ACE2	6M0J	Lan et al., 2020
**RBD**		
REGN10933, REGN10987	6XDG	Hansen et al., 2020
COR-101 (STE90-C11)	7B3O	Bertoglio et al., 2020
BRII-196	7BWJ	Ju et al., 2020
BD23	7BYR	Cao et al., 2020
B38	7BZ5	Wu Y et al., 2020
LY-CoV016	7C01	Shi et al., 2020
SR4, MR17	7C8V, 7C8W	Li et al., 2020
H014	7CAH	Lv et al., 2020
P2C-1F11, P2C-1A3	7CDI, 7CDJ	Ge et al., 2021
BD-604, BD-629, BD-368-2	7CH4, 7CH5, 7CHH	Du et al., 2020
COVA2-04	7JMO	Wu NC et al., 2020
COVA1-16	7JMW	Liu et al., 2020
S2A4, S304, S309, S2H14	7JVA, 7JW0, 7JX3	Piccoli et al., 2020
nb20	7JVB	Xiang et al., 2020
C002, C104, C110, C119, C135, C144	7K8S, 7K8U, 7K8V, 7K8W, 7K8Z, 7K90	Barnes et al., 2020
F52, F298	7K9Z	Rujas et al. 2020
S2E12	7K45	Tortorici et al., 2020
rCR3022	6XC7	Yuan et al., 2020
EY6A	6ZER	Zhou et al., 2020
CR3014-C8	7KZB	Rouet et al., 2021
MW06	7DPM	Jiang et al., 2021
VHH U, VHH V, VHH W	7KN5, 7KN6, 7KN7	Koenig et al. 2021
COVOX-45	7BEL	Dejnirattisai et al., 2021
**NTD**		
4-18, 5-24, 2-51, 1-87, 2-17	7L2E, 7L2F, 7L2C, 7L2D, 7LQW	Cerutti et al., 2021
4A8	7C2L	Chi et al., 2020
FC05	7CWS	Wang N. et al., 2021
S2L28, S2X33, S2M28	7LXX, 7LXY, 7LY3	McCallum et al., 2021a
COVOX-159	7NDC	Dejnirattisai et al., 2021
DH1052, DH1050.1	7LAB, 7LCN	Li et al., 2021
